# Acupuncture for Frequent Migraine: A Randomized, Patient/Assessor Blinded, Controlled Trial with One-Year Follow-Up

**DOI:** 10.1155/2015/920353

**Published:** 2015-04-28

**Authors:** Yanyi Wang, Charlie Changli Xue, Robert Helme, Cliff Da Costa, Zhen Zheng

**Affiliations:** ^1^TCM Research Program, Health Innovations Research Institute (HIRi), School of Health Sciences, RMIT University, Bundoora, VIC 3083, Australia; ^2^Department of Medicine, Royal Melbourne Hospital, Parkville, VIC 3052, Australia; ^3^School of Mathematical and Geospatial Science, RMIT University, Bundoora, VIC 3083, Australia

## Abstract

*Objectives*. This study aimed to evaluate the efficacy and safety of manual acupuncture as a prophylaxis for frequent migraine. *Methods*. Fifty frequent migraineurs were randomly allocated to receive 16 sessions of either real acupuncture (RA = 26) or sham acupuncture (SA = 24) during 20 weeks. The primary outcomes were days with migraine over four weeks, duration, and intensity of migraine and the number of responders with more than 50% reduction of migraine days. The secondary outcomes were the relief medication, quality of migraine, quality of life, and pressure pain thresholds. *Results*. The two groups were comparable at baseline. At the end of the treatment, when compared with the SA group, the RA group reported significant less migraine days (RA: 5.2 ± 5.0; SA: 10.1 ± 7.1; *P* = 0.008), less severe migraine (RA: 2.18 ± 1.05; SA: 2.93 ± 0.61; *P* = 0.004), more responders (RA: 19 versus SA: 7), and increased pressure pain thresholds. No other group difference was found. Group differences were maintained at the end of the three-month follow-up, but not at the one-year follow-up. No severe adverse event was reported. Blinding was successful. *Discussion*. Manual acupuncture was an effective and safe treatment for short-term relief of frequent migraine in adults. Larger trials are warranted.

## 1. Introduction

Migraine, a highly prevalent primary headache, affects 11%–16% of the population [1–3]. It is the 19th most prevalent disease that causes disability [4]. Its peak prevalence occurs in those aged between 25 and 55 years [5] and therefore affects a high percentage of adults in the productive phase of their lives. Over 90% of migraineurs report some level of functional impairment [5]. In the USA, migraineurs spend more than three million days in bed each month due to pain [6]. People with frequent migraine attacks report they are unlikely to return back to a normal level of biologic function [7].

To date, no “cure” exists for migraine. Although the pharmacotherapies provide some relief, they are associated with adverse events (AEs) such as low blood pressure, nausea, depression, drowsiness, and rarely renal damage. For this reason, 50% of chronic migraineurs and 27% of episodic migraineurs prefer nonpharmacotherapies and have used complementary therapies for migraine [8] including acupuncture [9]. A recent guideline developed by the National Institute for Health and Clinical Excellence of UK recommended a course of up to 10 sessions of acupuncture over 5–8 weeks for prophylactic treatment of migraine if both topiramate and propranolol are unsuitable or ineffective [10].

To date, clinical trials have shown that acupuncture is an effective alternative treatment for tension-type headache [11] and chronic headache [12]. The role of acupuncture for managing migraine headache, however, remains uncertain. There are a number of methodological issues associated with existing acupuncture trials for migraine, such as small sample size, inappropriate choice of instruments for outcome measure, or nonadherence to the International Headache Society Classification of migraine headaches for subject selection. Furthermore, a systematic review [13] found that the majority of trials did not devise an acupuncture protocol that reflects the practice of acupuncture. Moreover, frequent migraine with more than 5 attack days per month has not been studied specifically. Most studies measured the long-term effect of acupuncture within 2–6 months after the end of treatment. The effect at one year after acupuncture trials is unknown. Meanwhile, pressure pain threshold (PPT), reflecting the individual's sensitivity to pain, and the relationship between migraine and PPTs have not been fully understood [14, 15], although lower PPTs have been observed in tension-type headache sufferers [16, 17].

The present trial aimed to determine the short- and long-term effects and safety of acupuncture, compared with sham acupuncture for migraine sufferers who have headache more than 5 days per month.

## 2. Methods

### 2.1. Participants

All participants were volunteers suffering from migraine and recruited from the greater Melbourne area using a series of media releases and advertisements. Those who met the inclusion criteria of migraine according to the International Headache Society (IHS) [4], aged between 18 and 80 years, reported a current history of migraine for at least 12 months, and had a minimum of five days with migraine per four weeks were included. Patients were excluded if they were currently pregnant or had malignancy; if they had experience of acupuncture treatment in the face, the hands, the legs, or the front of the body in the previous six months; if they had a history of head injury or whiplash; if they had a severe arrhythmia or heart failure, brain tumor, or epilepsy; if they had hemophilia; if they had participated in another clinical trial in the past six months; if they had tension-type headache more than six days a month; if they were unable to distinguish between migraine attacks and tension-type headache, or if they did not comprehend English.

### 2.2. Randomization

After a four-week baseline, eligible participants were randomly allocated to real acupuncture (RA) or sham acupuncture (SA) groups. Block randomization was used, eight participants in each block, with a 1 : 1 ratio. An independent researcher prepared a computer-generated sequence of random numbers and processed the randomization. The opaque sealed envelopes were stored in a locked cabinet, and each block of envelopes (up to eight) was hand out to let the next eligible participant pick an envelope from the block. Participants were informed this being a randomized trial that compared the effect of real acupuncture with that of sham treatment without being told the block or the block size. They were also informed that sham treatment produced minimal effect in addition to the placebo effect. Only the treating acupuncturist was aware of the treatment allocation. Furthermore, independent assistants in charge of data entry or assessment were blinded to the treatment allocation. During the treatment period, any discussion related to treatment between the participants and the acupuncturist was restricted to a minimum of necessary explanations in order to ensure the success of the blinding procedure. After the first week of treatment, credibility of the acupuncture procedure was assessed with a questionnaire.

### 2.3. Interventions

During the 20-week treatment period, a total of 16 treatment sessions were delivered to participants. This occurred twice per week for four weeks (eight sessions) followed by once per week for another four weeks (four sessions), then once every two weeks for four weeks (two sessions), then once per month for another two months (two sessions). The location of acupoints adopted in the present study followed the Standard Acupuncture Nomenclature published by the World Health Organization [18]. The same acupuncturist, who completed a five-year bachelor degree in acupuncture, had more than three years of clinical experience, and was registered with the Chinese Medicine Registration Board of Victoria, Australia, performed all RA and SA treatment consistently throughout the trial. Besides the use of relief medications, no other concurrent interventions were permitted during the trial period.

A semistandardised acupuncture treatment protocol was used for both groups. This protocol consisted of a set of mandatory acupoints that were used for all participants and a set of supplementary acupoints that were selected based on individual diagnosis of Chinese medicine syndromes of migraine (Table 1). The selection of supplementary acupoints was flexible to meet the limit of needle number, which is 9–12 needles in total for each session of treatment. Needles used in both groups were 0.25 mm in diameter and either 30 mm or 40 mm in length (Hwato, Suzhou Medical Instrument Factory, China) according to the location of the acupoints.

For RA, needles were inserted transversely, obliquely, or perpendicularly to a depth of 10–30 mm depending on the specific locations of acupoints. De Qi sensation was induced. Needles were retained for 25 minutes, with further stimulation given every 10 minutes.

For SA, combined insertion and noninsertion procedures were used (Table 2).

### 2.4. Outcome Measures

The type of outcome measures and when they were measured are listed in Table 3. The primary outcome measures included the frequency, duration and intensity of migraine. The intensity of migraine was measured using a 0–10 Visual Analogue Scale (VAS) and a Six-Point Likert Scale. All of above were documented by participants in a headache diary daily throughout the baseline, treatment, and follow-up periods. Another primary outcome measure was the percentage of patients with more than 50% reduction in the number of days with migraine attack at the end of treatment.

The secondary outcome measures included the relief medication usage for migraine, the severity and quality of migraine [19], and quality of life [20]. Additionally, PPTs were measured in a standard sequence at 11 sites with 1 kg/cm^2^ force [21], before the first real or sham acupuncture treatment and after the last treatment of the trial (Table 4). Within each session, the PPT of each site was measured twice, and the mean of the two measurements represented the PPT value for that site. PPT was measured by an assessor blinded from the group allocation using a handheld pressure algometer (Wagner, Electronic Engineering Corporation of India). The apparatus consists of a 1 cm in diameter hard rubber tip, attached to the plunger of a pressure (force) gauge. The dial of the gauge is calibrated in kg/cm^2^.

### 2.5. Statistical Analysis

Intention to treat (ITT) analyses were performed for all the outcome measurements of post treatment and follow-up I. Per protocol (PP) analyses of outcome measures were conducted with follow-up II data. Chi-square or* t*-tests were used to assess the comparability of the sociodemographic characteristics, the number and percentage of AEs, and baseline headache data between the two groups. Repeated measures of General Linear Model (ANOVA) were used to test the short-term effects of acupuncture, including the main effect of treatment group and group by time interaction. Paired-samples* t*-test and independent-sample* t*-tests with Bonferroni correction were used for post hoc analyses. The long-term effects were analysed using paired sample* t*-tests.

The significance level used was *α* = 0.05. If the multiple comparison procedures were conducted in one outcome at different time points, significance level was adjusted by dividing 0.05 with the number of comparisons.

Any missing data in the headache diary, MPQ, or PPT was replaced by using the “Missing Value Analysis” function under “Analysis function” in the Statistical Package for the Social Sciences (SPSS, version 15.0 for windows) software program. MSQOL missing data was dealt with according to the instruction manual.

### 2.6. Sample Size Calculation

The sample size was calculated based on an acupuncture trial on migraine [22]. The mean frequency (standard deviation) of headaches in the treatment and waiting list groups were 1.5 (1.2) and 2.3 (1.1). Using those data, it was estimated that the current trial required a sample size of 33 per group to reach a statistical power of 80%. As an intention to treat analysis was used, no additional participants were needed to compensate for the dropouts. Consequently, a total of 66 participants were needed for this study.

## 3. Results

**Figure 1:**
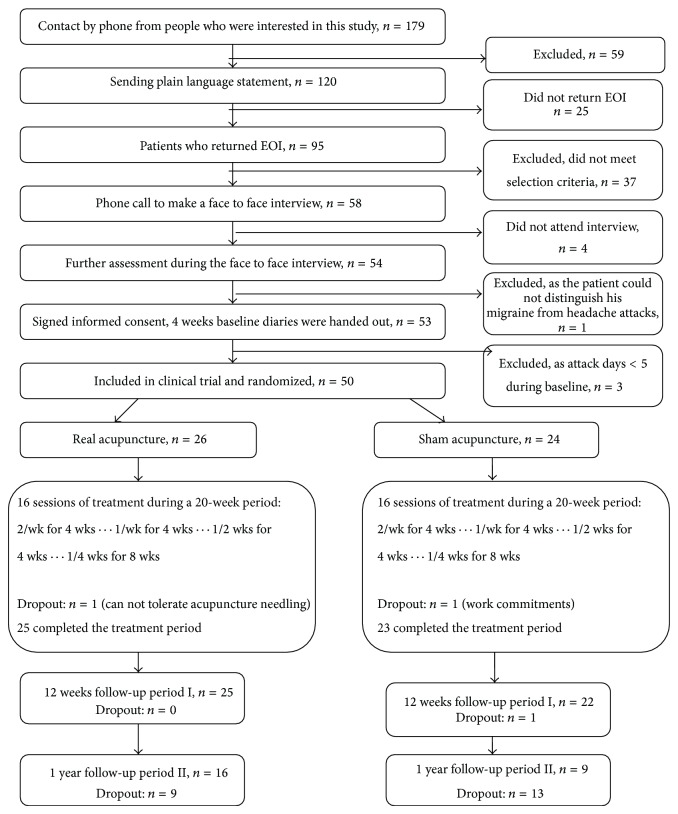
Number of participants in different stages of trial. EOI: expression of interest.

Figure 1 illustrates the trial process and number of participants at each stage. Fifty participants out of 179 enquires were enrolled and randomly allocated into either the RA (*n* = 26) or the SA (*n* = 24) groups. Forty-eight participants completed the 20-week treatment, with one withdrawing from each treatment group, due to disliking acupuncture needling sensation (RA group) and work commitments (SA group), respectively. For unknown reasons, a participant of the SA group withdrew during the 12 weeks follow-up period. The dropout rate for the treatment period was low (4%) for each group. On average, participants from the RA and SA received 15.4 and 15.6 treatment sessions over 20 weeks, respectively. However, only 25 out of 47 participants' data were available for the one-year long-term effect analysis (follow-up period II). Based on the screening result of 50 participants, the most common accompanying symptoms with headache were light sensitivity (84%), nausea (82%), unilateral headache (80%), sound sensitivity (66%), pulsating quality (64%), aggravation by or causing avoidance of routine physical activity (64%), and vomiting (50%). The demographic and headache features (Table 5) were comparable at baseline and are representative of the characteristics of migraine in prevalence studies [5].

### 3.1. Primary Outcomes of Efficacy

**Figure 2:**
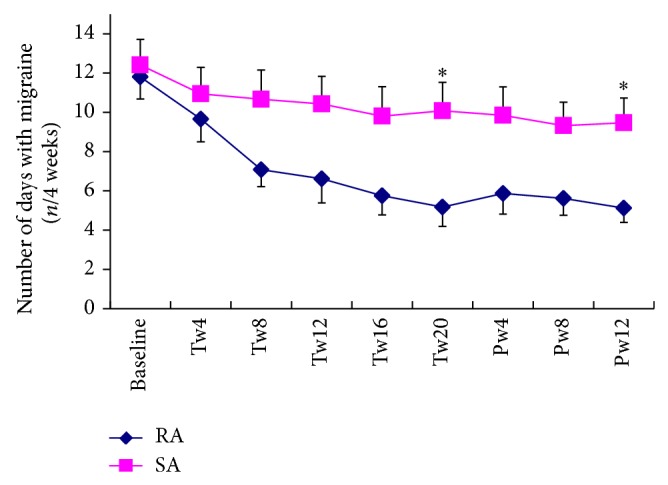
The number of days with migraine per four weeks in each group across all treatment time points (mean and SE). Tw4, Tw8, Tw12, Tw16, and Tw20 correspond to the treatment weeks 1–4, weeks 5–8, weeks 9–12, weeks 13–16, and weeks 17–20, respectively; Pw4, 8, and 12 correspond to the posttreatment weeks 1–4, weeks 5–8, and weeks 9–12; ∗ indicated that at that point in time the significant difference between two groups was detected.

**Figure 3:**
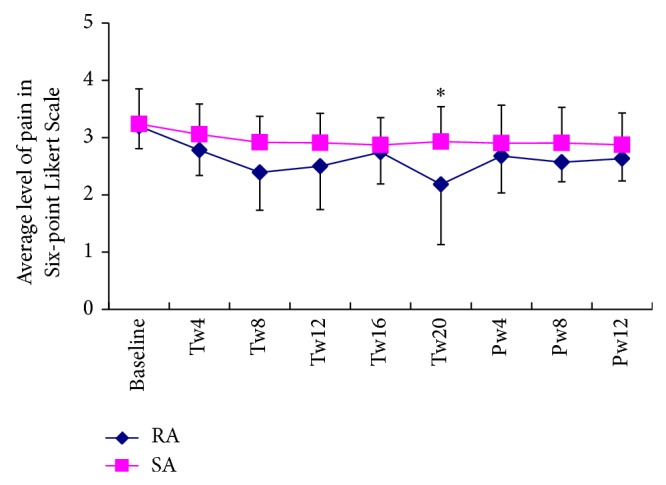
The time course of the average pain measured with a six-point Likert scale over the five treatment phases (mean and SD). Tw4, Tw8, Tw12, Tw16, and Tw20 correspond to the treatment weeks 1–4, weeks 5–8, weeks 9–12, weeks 13–16, and weeks 17–20, respectively; Pw4, 8, and 12 correspond to the posttreatment weeks 1–4, weeks 5–8, and weeks 9–12; ∗ indicated that at that point in time, the significant difference between two groups was detected.

The number of days with migraine (migraine days) was significantly reduced in both groups over the 20-week treatment period [*F*(5, 240) = 18.4, *P* < 0.001] and the reduction was greater in the RA group than in the SA group [*F*(5, 240) = 4.5, *P* = 0.002]. Post hoc analysis revealed that the RA group has less migraine days than SA group did, at the end of treatment and 3-month follow up (Table 6). The outcomes for pain severity and number of days with migraine across the whole treatment and 3-month follow-up period are illustrated in Figures 2 and 3. The group difference maintained at the end of 3-month follow-up (*P* = 0.005). When we used the prespecified cut-off point of 50% reduction in the number of days with migraine to define a responder, 19 individuals in the RA group documented a response at the end of treatment period whereas only seven participants in the SA group did (*P* = 0.002). A similar response was shown at the end of the 3-month follow-up (*P* = 0.034). Meanwhile, the RA group experienced a faster reduction of migraine pain (average) when compared with the SA group [*F*(5, 240) = 3.14, *P* = 0.02] (Figure 3).

### 3.2. Secondary Outcomes of Efficacy

There was no group difference in McGill pain questionnaire data except for PRI-emotional, which was better in the RA group (Table 7). With respect to the quality of life assessed by MSQOL, there were statistically significant time effects on function-restrictive (FR) [*F*(5, 240) = 8.6, *P* < 0.001] and emotional function (EF) [*F*(5, 240) = 10.8, *P* < 0.001] and treatment group by time interaction on role function-preventive (FP) [*F*(5, 240) = 3.0, *P* = 0.023] and EF [*F*(5, 240) = 596, *P* < 0.001] with the RA group showing a faster improvement on FP and EF than the SA group did.

At the end of treatment, more participants in the RA group used less pain killers as relief medication than in the SA group (*P* = 0.004), and participants in the RA group experienced a quicker reduction (treatment group by time interaction [*F*(5, 240) = 2.5, *P* = 0.064]), although both groups reduced their medication during the treatment period (time effects [*F*(5, 240) = 7.1, *P* < 0.001]). However, there were neither time effects [*F*(5, 240) = 1.5, *P* = 0.18] nor treatment group by time interaction [*F*(5, 240) = 0.9, *P* = 0.52] on the pill count, including prophylactic and acute-pain control medication.

### 3.3. Pressure Pain Threshold

**Figure 4:**
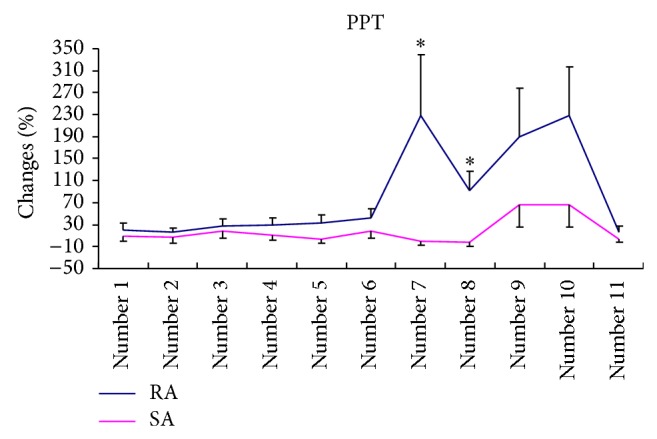
The percentage change of PPTs at 11 sites in the two groups after the treatment (mean and SE, RA, *n* = 26, and SA, *n* = 24). ∗ indicates that at the end of treatment, the mean percentage changes in PPTs of sites Numbers 7 and 8 in RA were significantly larger than those in SA.

Percentage changes in PPT varied significantly among the 11 sites. Generally, the two groups showed similar trends in the PPTs changes across the sites [*F*(10, 480) = 2.4, *P* = 0.11] (Figure 4). Post hoc analyses were conducted using Independent-sample* t*-tests. After the treatment, PPTs either were not changed or demonstrated a very small increase at all sites in both groups except for those at sites Numbers 7 and 8 (left and right EX-HN5), located at the temporal region of the head, at which sites the RA group reported significantly higher PPTs than did the SA group.

In the RA group, mean increases in PPTs ranged from 15.84% at Number 11 (EX-HN3) to 229.48% at Number 7. In the SA group, the range was from a decrease of 0.66% at Number 7 to an increase of 66.86% at Number 9 (left ST-6). No significant correlations were detected between changes in frequency, duration, and intensity of migraine with changes of PPTs at all 11 selected sites.

### 3.4. Efficacy of Acupuncture at One-Year Follow-Up

At the end of the one-year follow-up period, only 25 out of 47 participants completed the headache diary, consisting of 16 from the RA group and nine from the SA group. No statistically significant group difference was detected in any outcome measures (Table 7).

### 3.5. Safety

Thirty-seven AEs were reported out of 400 sessions (9.25%) in the RA group and 14 of 374 sessions (3.74%) in the SA group (Table 8). All AEs were reported as mild or moderate. None of the AEs required medical interventions. One participant in the RA group experienced severe tingling sensation after a needle was inserted into Hegu (LI4) on the right hand. This participant described tingling which could be felt on the right side of the face and which lasted for one hour and disappeared after some rest. She withdrew from the study.

Credibility of the blinding was assessed at the end of the first treatment week after two sessions. All 50 participants completed a three-item questionnaire. The credibility of sham needling at the early stage of the trial was successful with no statistically significant difference between the two groups (*P* = 0.88). Seventeen participants could not tell which group they were in and did not select any reason. The majority of the remaining 33 participants made a guess based on the result of the treatment or the manner, attitude, or communication with of the acupuncturist in the trial. There was no group difference in the reasons (details see Table 9).

## 4. Discussion

The present trial showed that acupuncture was effective in reducing migraine days, as well as effecting a reduction of medication consumption and improvement in quality of life, when compared with sham acupuncture. The effect lasted up to three months but seems to have ceased one year after the termination of the treatment. However this conclusion about a lack of the long-term is based on data from less than 50% of the participants who returned the diary. There were no serious adverse events that necessitated withdrawal of participants from the trial. The incidence and severity of minor adverse effects were comparable between the two groups. The participants were properly blinded. The above results demonstrated that manual acupuncture can be an effective and a safe prophylaxis for frequent migraine sufferers. Because the findings of the present study were based on self-selected community-based participants; the results discussed here are limited to this specific group.

### 4.1. Strengths

The current RCT is unique when compared with previous studies of acupuncture for migraine in the following four aspects. First, these participants all experienced a minimum of five days of migraine or more during the four-week baseline period. People with frequent migraine attacks were chosen because the effect of acupuncture on this group of population has not been well studied [23, 24]. Other studies have typically included patients having two to five or two to eight migraine attacks per month [22, 25–27]. The mean days with migraine in those publications ranged from 5 to 6.1 days per four weeks [22, 28]. The mean attack days at baseline in the current study were about 12 days, much higher than previous published studies. Most of the studies reported from 3.7 to 6.4 migraine days per month after acupuncture treatment [22, 25, 27], which consists with 5.17 migraine days per month after acupuncture treatment in our study.

Second, the current study has the longest treatment period (20 weeks) of any in the literature and incorporated gradual decrease in treatment frequency. This treatment regime reflects how acupuncture is practiced in a clinical setting and has been shown to be effective in one positive acupuncture trial for osteoarthritis in the knee [29]. The current study also has the longest follow-up period at one year, except for two studies on chronic headache [30, 31].

Third, we adopted an innovative sham acupuncture design. In published acupuncture trials, it is often difficult to establish a true placebo intervention, as sham acupuncture is not an inert treatment [32] and may produce nonspecific effects [33], such as the analgesic effects produced via diffuse noxious inhibitory control (DNIC) by simply piercing the skin [32]. Even so, many previous studies used shallow or deep needling into sham points [26, 31, 34], and the sham points are often within the same region in which the points used for real acupuncture treatment or where the diseases reside. A review on methodology of sham acupuncture found that placing needles in the same dermatome or myotome of the disease produced a strong therapeutic effect [35]. Once the needles are inserted, the spinal “gate control” system could be activated thereby producing pain relief in the same and adjacent spinal nerve segments. Our unique sham acupuncture procedure employed a combined shallow insertion procedure in distal area to enhance the credibility of sham intervention and a noninsertion procedure on the points in the cranial area to minimize the nonspecific effect of acupuncture on migraine. This procedure seems to have been successful in this study.

Forth, we measured PPT on 11 sites on the scalp and face during nonmigraine days in addition to pain measurement. It is interesting to note that the PPTs at only two sites, Numbers 7 and 8, statistically significantly increased more than those in the SA group. These two sites are located on either side of the m. temporalis, over the superficial temporal artery and vein and near the second and third branches of the trigeminal nerve. The temporal artery [36] and trigeminal nerve are associated with the development of migraine [37]. The neurovascular theory of migraine considers that vasodilatation activates stretch receptors in the wall of the temporal artery, stimulating the perivascular trigeminal nerves and leading to neurogenic inflammation. The inflammation in turn activates the trigeminal nuclei and further enhances the sensitivity of the nervous system prior to a migraine attack [38]. Acupuncture has been shown to increase pain threshold in many other studies [39, 40]; however the relationship between enhanced PPTs in the temporal area and sensitivity of stretch receptors in the wall of the temporal artery is unknown. It is possible that acupuncture reduces the sensitivity of these receptors and therefore prevents the activation of trigeminal nerves. This hypothesis should be explored in the future as it might contribute to our understanding of the antimigraine mechanisms of acupuncture. Meanwhile temporal regions has been considered to be the most reliable site when the repeatability of PPT was assessed at a few sites, including 13 sites located in anterior, upper, posterior, and temporal areas of the head [41].

Finally, in order to ensure the credibility of the sham acupuncture, participants with limited acupuncture experience were recruited, and it was demonstrated that they could not identify real from sham acupuncture according to past experience.

### 4.2. Limitation

The main limitation is the small sample size. Inadequate sample size can skew findings [42] and this has been a common problem for acupuncture trials [43, 44]. The originally targeted sample size for the present study was a total of 66 participants, based on a migraine study which achieved statistical significance between acupuncture and waiting list groups analysed for frequency of headaches. In the end, our study only managed to recruit 50 participants. There are several reasons, which may contribute to the difficulties with recruiting participants. First of all, with the increasing popularity of acupuncture, it is difficult to enroll participants who have no or limited previous acupuncture experience. Surveys have revealed that headache sufferers accounted for approximately 10% of visits to acupuncturists in USA [45] and more than 25% in Germany [46]. Secondly, our criterion of recruiting only participants who had five days or more of migraine per month excluded many interested migraine sufferers who had a lower frequency of attack, thus limiting our recruitment capability. Finally, the long-treatment period might also have been an obstacle or impediment. Some people were unwilling and or unable to commit themselves for such a long period of time. The Oxford Pain Validity Scale, which is designed to assess the quality of clinical trial for pain conditions, has defined trials with 40 or more participants per group as a satisfactory sample size. Nevertheless, a small sample size is still possible if more disease-specific primary outcome measures, such as frequency or intensity of migraine headaches, are chosen [44]. Consequently, in the current study, there was still sufficient power to detect significant changes in frequency and intensity of migraine. Furthermore, we conducted the skewness tests for all primary and secondary outcomes and found that some of data were not normally distributed such as the data of pill count. Nonparametric tests were applied to those data. However, the results were not different from those of parametric tests. Statisticians believe when dealing with a sample size larger than 30 or 40, violation of the normality assumption should not cause major problems to the data analysis [47] and parametric procedures could be used [48]. Our findings are therefore not affected by the type of statistical analysis method used.

Another potential limitation is that frequent migraine sufferers might have a higher expectation of acupuncture than those less affected by the condition. Previous studies have shown that the expectation caused greater treatment activation than skin prick [49]. A low back pain study showed that patients with a high expectation of acupuncture may have a better outcome than those with a low expectation [50]. Another study that examined four acupuncture trials for painful conditions reported a significant association between better improvement and a higher expectation [51]. In the current study all participants were volunteers. 62% of participants joined the study because they learnt from others' experience that acupuncture was helpful. Although not assessed in the current study, expectation is less likely to be the reason underlying the group differences. The participants were successfully blinded from the treatment allocation as indicated by the credibility questionnaire, and the randomization distributed participants equally to RA and SA groups. This factor should be assessed in all future studies. A newly developed Acupuncture Expectancy Scale may assist future research [52].

### 4.3. Comparison with Other Studies

The findings of the current study on migraine days demonstrated that the RA was significantly better than SA, which is supported by some studies [24, 32, 53]. However, other studies have reported no difference between RA and SA [22, 25, 27]. The positive results of the current study could be due to a higher frequency of migraine per month at baseline than reported in other studies. A study with 284 migraineurs and 17 tension-type headache patients found that obvious improvement in the number of days with migraine appeared in the participants with more than four days with headache per month [12]. Other reasons could be due to our long treatment regime, a gradual reduction in treatment frequency, and an innovative sham acupuncture design as discussed above.

Although RA acupuncture reduced the duration of each migraine attack in the RA group, there was no group difference on this measure in the current study. Two studies conducted by Alecrim-Andrade and her colleagues demonstrated that acupuncture reduced the total hours in pain per four weeks [28, 53]. However, these studies employed the widely used measurement instrument, the Headache Index [54, 55]. Results obtained from the Headache Index should be interpreted with care because they combined the duration of each attack with the number of attacks. It is important to understand that decrease in the frequency of attack alone can contribute to a reduction of the total number of hours in migraine per four weeks. This assessment method is different from our single measure of hours per attack. The IHS clinical trials committee has also commented that a decrease in total hours of pain is often due to a decrease in the frequency of attacks [56]. We consider that our single measure method better reflects the nature of migraine days and the effect of acupuncture. Currently there are only a few studies that have used the same single measurement of duration of migraine as in our study [57, 58].

## 5. Conclusion

Acupuncture can be used as alternative and safe prophylaxis for frequent migraine. Our recommendation is that practitioners treat migraine sufferers twice per week for at least eight weeks. Reduced medication usage is expected during acupuncture treatment. Future studies need to assess if regular follow-up treatments, perhaps at a monthly or bimonthly interval after an initial three-month hiatus, might provide long-term prophylaxis for this group of patients.

## Figures and Tables

**Table 1:** Acupoints selection for the real acupuncture group.

Syndromes	Mandatory acupoints (unilateral)	Supplement acupoints (bilateral)
Ascending hyperactivity of liver yang	Fengchi (GB20, bilateral) Taiyang (EX-HN5) Shuai Gu (GB8) Hegu (LI4) Unilateral: points on the side of current migraine or points on the side of the last migraine episode, if no current migraine.	Baihui (DU20), Xingjian (LR2), Taichong (LR3), Taixi (KI3), Xuanzhong (GB39), and Sanyinjiao (SP6)
Deficiency of both Qi and blood		Baihui (DU20), Shang Xing (DU23), Zusanli (ST36), and Sanyinjiao (SP6)
Wind phlegm blocking the meridians		Feng Long (ST40), Zhongwan (CV12), and Yinlingquan (SP9)
Blood stasis		Sanyinjiao (SP6), Xuehai (SP10), and Ashi point

**Table 2:** Method of sham acupuncture.

	Local sham points on the scalp, face, and neck	Distal sham points on the four extremities
Technique	Noninvasive, using a blunted cocktail-stick	Minimal acupuncture, 2 mm depth insertion

Sham point	1-2 cm away from the real acupoints	1-2 cm away from the real individual distal supplementary points according to the syndrome differentiation

Stimulation	The stick was tapped	No needling manipulation, avoid De Qi

**Table 3:** Outcome measures used in the trial.

Outcome measures	Instruments	Baseline (4 weeks)	Treatment (20 weeks)	Follow-up (phase 1^*^ and phase II^#^)
Primary outcomes				
Frequency of migraine	Headache diary	Daily for 4 weeks	Daily for 20 weeks	Phase I: daily for 12 weeks and this is between 21st week and 32nd week after randomization. Phase II: daily for 4 weeks and this is between 68th week and 72nd week after randomization.
Duration of migraine	24-hour Visual Analogue Scale (VAS)			
Intensity of migraine	0–10 VAS and a Six-Point Likert Scale			
Response rate	Percentage of patients with more than 50% reduction in the number of days with migraine attack per four weeks	Calculated based on the frequency data and reported on weekly basis		

Secondary outcomes				
Relief medication usage	Pill count and the Medication Quantification Scale	Daily for 4 weeks	Daily for 20 weeks	Phase I: daily for 12 weeks and this is between 21st week and 32nd week after randomization. Phase II: daily for 4 weeks and this is between 68th week and 72nd week after randomization.
Severity and quality of migraine	McGill pain questionnaire [19]	At the last week of baseline	At the 4th, 8th, 12th, 16th, and 20th week after randomization	Phase I: at the 24th, 28th, and 32nd week after randomization. Phase II: at the 72nd week after randomization.
Quality of life	Migraine Specific Quality of Life questionnaire [20]			
Pressure pain threshold	A handheld pressure algometer	Before the 1st treatment	After the last treatment	

^*^Phase I follow-up period: 3 months from the end of the treatment.

^#^Phase II follow-up period: one year from the end of the treatment.

**Table 4:** The points used for testing pressure pain threshold.

Number 1	Left	2 cm inferior to the external occipital protuberance and 2 cm lateral to the midline
Number 2	Right	

Number 3^*^	Left	GB20: in a depression between the upper portions of the sternocleidomastoid muscle and the trapezius
Number 4^*^	Right	

Number 5	Left	2 cm lateral to GV20, which locates on the head, 5 cun^#^ directly above the midpoint of the anterior hairline, at the midpoint of the line connecting the apexes of both ears
Number 6	Right	

Number 7^*^	Left	EX-HN5: in the temple region, in a depression about 1 cun posterior to the midpoint between the lateral end of the eyebrow and the outer canthus of the eye
Number 8^*^	Right	

Number 9	Left	ST6: one finger width anterior and superior to the angle of the mandible at the belly of the masseter muscle when teeth clenched
Number 10	Right	

Number 11		EX-HN3: at the midpoint of the line connecting the medial ends of the eyebrows

^#^Cun is a Chinese word that translates as “anatomical inch”. The length of one cun is individualized. For instance, the distance from the eyebrow to the forehead hairline is defined as 3 cun.

^*^Also indicates the acupoints needled in the RA group.

**Table 5:** Comparisons of demographic variables at baseline.

Variables	RA (*n* = 26)	SA (*n* = 24)	*P* value
Demographic data			
Age (years) mean (SD)	41.6 (14.9)	43.8 (13.4)	0.58
Migraine history (years) mean (SD)	18.4 (12.7)	21.1 (13.3)	0.47
Gender *n* (%)			
Female	18 (75%)	19 (73.1%)	0.88
Acupuncture experience *n* (%)			
No	10 (41.7%)	14 (53.9%)	0.39
Yes	14 (58.3%)	12 (46.1%)	
Marital status *n* (%)			
Partnered	17 (70.8%)	17 (65.4%)	0.68
Single	7 (29.2%)	9 (34.6%)	
Education level (*n*)			
H: university or higher	10	12	0.52
S: 9 or more years of formal education	12	14	
L: less than 9 years	1	0	
M: missing data	1	0	
Type of migraine *n* (%)			
MO: migraine without aura	16 (32%)	13 (26%)	0.46
MA: migraine with aura	3 (6%)	6 (12%)	
Both: MO and MA cooccurrence	5 (10%)	7 (14%)	

Outcomes measures^#^			
Migraine days (number of days with migraine per 4 weeks)	11.8 (5.8)	12.4 (6.4)	0.73
Duration (hours /attack)	9.0 (3.6)	8.9 (4.8)	0.91
Highest pain-VAS	6.0 (1.1)	5.3 (2.0)	0.11
Lowest pain-VAS	3.1 (1.8)	2.7 (1.9)	0.39
Average pain-VAS	4.6 (1.4)	4.0 (2.0)	0.21
Severity of pain (six-point Likert scale)	3.2 (0.4)	3.2 (0.6)	0.79
McGill			
PRI-S	18.3 (8.9)	21.3 (7.5)	0.20
PRI-A	5.4 (2.7)	6.7 (3.2)	0.14
PRI-E	3.5 (1.3)	3.6 (1.1)	0.73
PRI-M	6.7 (3.0)	7.6 (3.2)	0.34
Total	33.9 (13.4)	39.0 (13.2)	0.19
MSQOL			
FR	54.2 (17.2)	46.7 (18.9)	0.15
FP	71.0 (19.0)	61.7 (20.6)	0.10
EF	54.7 (24.8)	48.5 (24.2)	0.40
Medication			
MQS	93.8 (81.1)	87.0 (110.2)	0.81
Pill count	13.50 (20.27)	7.27 (13.50)	0.54
Number of participants who took pain killers	22	23	0.71
Number of participants who took specific antimigraine drugs	4	3	0.60
Number of participants who took prophylactic drugs	12	11	0.59

RA: real acupuncture group; SA: sham acupuncture group; ^#^Clinical data were summarized as mean (SD); PRI-S: sensory components; PRI-A: affective components; PRI-E: evaluative components; PRI-M: miscellaneous components; FR: function-restrictive in Migraine Specific Quality of Life questionnaire; FP: function-preventive in Migraine Specific Quality of Life questionnaire; EF: emotional function in Migraine Specific Quality of Life questionnaire; PPT: pressure pain threshold; MQS: Medication Quantification Scale.

**Table 6:** Primary outcome measurements.

	After treatment (*ITT*)				At three months follow-up period I (*ITT*)				One-year follow-up period II (*PP*)			
	RA mean (SD)	SA mean (SD)	95% CI for difference	*P* value	RA mean (SD)	SA mean (SD)	95% CI for difference	*P* value	RA mean (SD)	SA mean (SD)	95% CI for difference	*P* value
	*n* = 26	*n* = 24			*n* = 26	*n* = 24			*n* = 16	*n* = 9		
Migraine days (number of days with migraine per 4 weeks)	5.2 (5.0)	10.1 (7.1)	(−8.5, −1.3)	0.008^*^	5.1 (3.7)	9.5 (6.2)	(−7.2, −1.4)	0.005^*^	11.3 (5.5)	12.3 (5.6)	(−5.8, 3.7)	0.660
Duration (hours/attack)	6.0 (3.3)	8.0 (4.5)	(−4.3, 0.2)	0.073	8.2 (2.8)	8.81 (4.1)	(−2.6, 1.3)	0.520	7.51 (2.6)	7.61 (3.5)	(−2.6, 2.4)	0.930
Highest pain-VAS	5.0 (1.8)	4.5 (1.9)	(−0.2, 1.6)	0.350	4.8 (1.4)	4.66 (2.0)	(−0.9, 1.1)	0.800	5.81 (1.0)	4.91 (2.4)	(−1.8, 1.2)	0.710
Lowest pain-VAS	2.3 (1.9)	2.5 (1.7)	(−1.2, 0.9)	0.820	2.6 (1.1)	2.62 (1.7)	(−0.9, 0.7)	0.860	2.77 (1.5)	3.04 (2.1)	(−1.0, 2.8)	0.310
Average pain-VAS	3.0 (1.8)	3.2 (1.8)	(−1.3, 0.8)	0.650	3.9 (1.0)	3.60 (1.9)	(−0.6, 1.7)	0.480	4.21 (1.1)	3.39 (2.1)	(−0.8, 2.5)	0.290
Severity of pain (six-point Likert scale)	2.2 (1.1)	2.9 (0.6)	(−1.2, −0.3)	0.004^*^	2.6 (0.4)	2.9 (0.6)	(−0.5, 0.0)	0.087	3.11 (0.5)	3.3 (0.7)	(−0.7, 0.3)	0.420
Responder^+^	19 (73.1%)	7 (29.2%)		0.020^#^	17 (65.4%)	5 (20.8%)		0.034^#^	1 (6.3%)	0 (0%)		

^*^Statistical significance assessed at 0.05/4 = 0.0125 (Bonferroni correction) for both intention to treat (*ITT*) and per protocol analysis (*PP*).

^+^Responder: participants with 50% or greater reduction in days with migraine.

^#^Indicates statistical significance assessed at 0.05.

**Table 7:** Secondary outcome measurements.

	After treatment (*ITT*)				At three months follow-up period I (*ITT*)				One year follow-up period II (*PP*)			
	RA mean (SD)	SA mean (SD)	95% CI for difference	*P* value	RA mean (SD)	SA mean (SD)	95% CI for difference	*P* value	RA mean (SD)	SA mean (SD)	95% CI for difference	*P* value
	*n* = 26	*n* = 24			*n* = 26	*n* = 24			*n* = 16	*n* = 9		
McGill												
PRI-S	13.1 (8.8)	15.8 (11.6)	(−8.6, 3.1)	0.350	11.1 (4.2)	14.1 (9.0)	(−7.1, 1.1)	0.150	21.6 (8.1)	19.7 (10.8)	(−6.0, 9.8)	0.620
PRI-A	3.96 (3.0)	4.8 (2.8)	(−2.5, 0.8)	0.320	3.3 (2.1)	4.4 (3.1)	(−2.6, 0.4)	0.163	6.5 (2.5)	6.2 (3.4)	(−2.2, 2.7)	0.820
PRI-E	2.08 (1.3)	2.8 (1.5)	(−1.53, .02)	0.056	1.6 (0.9)	2.6 (1.3)	(−1.6, −.38)	0.002^*^	3.6 (1.2)	3.8 (1.2)	(−1.2, 0.8)	0.700
PRI-M	5.6 (3.4)	6.1 (3.5)	(−2.47, 1.45)	0.60	5.5 (0.9)	5.9 (2.9)	(−1.9, 1.2)	0.651	7.3 (2.5)	8.1 (2.9)	(−3.2, 1.4)	0.440
Total	20.8 (15.7)	33.4 (17.4)	(−22.1, −3.2)	0.010^*^	15.0 (4.5)	22.4 (14.5)	(−13.5, −1.4)	0.017	38.9 (11.8)	37.8 (16.0)	(−10.4, 12.7)	0.843
MSQOL												
FR	72.2 (16.4)	58.0 (21.0)	(3.6, 24.9)	0.010^*^	74.2 (15.3)	56.3 (23.2)	(6.5, 29.2)	0.003^*^	46.4 (15.2)	50.1 (19.0, −0.5)	(−18.0, 10.6)	0.600
FP	77.1 (16.8)	68.3 (22.7)	(−2.7, 20.3)	0.13	80.8 (15.3)	63.1 (23.2)	(5.1, 30.2)	0.007^*^	62.0 (15.4)	64.3 (17.8)	(−16.3, 11.71)	0.740
EF	78.3 (19.3)	60.5 (25.6)	(4.98, 30.6)	0.007^*^	79.3 (18.7)	58.8 (27.3)	(7.1, 34.0)	0.002^*^	49.5 (17.0)	56.6 (21.0)	(−23.1, 8.8)	0.370
Medication												
MQS	20.8 (46.4)	68.9 (81.2)	(−85.4, −10.9)^*^	0.012	11.0 (24.3)	54.3 (62.4)	(−69.8, −16.8)	0.002^*^	78.2 (79.1)	75.1 (50.8)	(−59.5, 67.7)	0.920
Pill count	4.54 (12.21)	10.54 (19.31)	(−15.12, 3.11)	0.192	3.46 (9.84)	10.10 (17.63)	(−14.93, 1.64)	0.113	10.56 (14.81)	7 (9.90)	(−7.91, 15.03)	0.530
Number of participants who took pain killers	9	18	8.2^∧^	0.004^#^								
Number of participants who took specific antimigraine drugs	1	4	2.28^∧^	0.13								
Number of participants who took prophylactic drugs	7	9	.64^∧^	0.42								

^*^Indicates statistical significance assessed at 0.05/4 = 0.0125 (Bonferroni correction) for both intention to treat (*ITT*) and per protocol analysis (*PP*); ^#^indicates statistical significance assessed at 0.05; ^∧^indicates *χ* ^2^ value;  *PP*: indicates that the data are based on the per protocol analysis; PRI-S: sensory components; PRI-A: affective components; PRI-E: evaluative components; PRI-M: miscellaneous components; FR: function-restrictive in Migraine Specific Quality of Life questionnaire; FP: function-preventive in Migraine Specific Quality of Life questionnaire; EF: emotional function in Migraine Specific Quality of Life questionnaire; PPT: pressure pain threshold; MQS: Medication Quantification Scale.

**Table 8:** The adverse events reported by participants in each treatment group.

Type of event	RA (number of cases)	SA (number of cases )
Dizziness	4	3
Bruising	3	1
Pain	3	2
Cold and sweaty	8	5
Tingling	11	1
Recurrent headache	7	2
Mild spasm in the calf muscle induced by tapping on the thigh by the patients after the treatment	1	0
Total of AEs	37	14
Total of treatment sessions	400	374
Accidences per treatment	9.25%	3.74%

**Table 9:** The reason of guessing group allocation.

	RA group	SA group	Total	*χ* ^2^ value	*P* value
	*n* = 26	*n* = 24			
No selection (participants indicate they could not guess the group allocation)	9	8	17	1.88	0.76
Manner, attitude, or words of acupuncturist	3	4	7		
Manner, attitude, or words of the personnel in the clinic	5	6	11		
Results of the treatment	8	4	12		
Others	1	2	3		
Total	26	24	50		
